# Community-based sero-prevalence of hepatitis B and C infections in South Omo Zone, Southern Ethiopia

**DOI:** 10.1371/journal.pone.0226890

**Published:** 2019-12-30

**Authors:** Adugna Endale Woldegiorgis, Woldearegay Erku, Girmay Medhin, Nega Berhe, Mengistu Legesse

**Affiliations:** 1 Aklilu Lemma Institute of Pathobiology, Addis Ababa University, Addis Ababa, Ethiopia; 2 School of Medicine, College of Medicine and Health Sciences, Dire Dawa University, Dire Dawa, Ethiopia; 3 Department of Microbiology, Immunology and Parasitology, School of Medicine, College of Health Sciences, Tikur Anbessa Hospital, Addis Ababa University, Addis Ababa, Ethiopia; Centers for Disease Control and Prevention, UNITED STATES

## Abstract

**Background:**

Hepatitis B Virus (HBV) and Hepatitis C Virus (HCV) are the leading causes of liver-related morbidity and mortality throughout the world. The magnitude of HBV and HCV infections in Ethiopia has not been well studied at community level. This study aimed at investigating the sero-prevalence and associated risk factors of HBV and HCV among HBV unvaccinated community members in South Omo Zone, Southern Ethiopia.

**Methods:**

A community-based cross-sectional study was conducted in three districts from March to May 2018. Structured questionnaire was used to collect relevant clinical and socio-demographic data. Three milliliter of blood sample was collected from each study participant and screened for HBV and HCV using one step hepatitis B surface antigen (HBsAg) test strip and one step HCV test strip, respectively. Samples found positive for HBsAg were further tested using immunoassay of Alere Determine^TM^ HBsAg (Alere Inc., USA). Data were analyzed using SPSS version 25.0.

**Results:**

A total of 625 (51.4% males, age 6–80 years, mean age ± SD = 30.83 ± 13.51 years) individuals participated in the study. The sero-prevalence for HBV infection was 8.0% as detected using one step HBsAg test strip, while it was 7.2% using Alere Determine^TM^ HBsAg test. The sero-prevalence for HCV infection was 1.9%. Two (0.3%) of the participants were seropositive for both HBV and HCV infections. High sero-prevalence for HBV infection was associated with weakness and fatigue (AOR = 5.20; 95% CI: 1.58, 17.15), while high sero-prevalence of HCV infection was associated with age group between 46 and 65 years (AOR = 13.02; 95% CI: 1.11, 152.41).

**Conclusion:**

This study revealed higher-intermediate endemicity level of HBV infection and low to intermediate endemicity level of HCV infection in the study area. Clinical symptoms like weakness and fatigue were found to be indictors for HBV infection, while individuals in the age group between 46 and 65 years were at higher risk for HCV infection. Provision of community- based health education; vaccination, mass screening and providing treatment would have utmost importance in reducing the transmission of these diseases in the present study area.

## Introduction

Hepatitis, inflammation of the liver, can be caused by infectious and non-infectious agents such as viruses, bacteria, fungi, parasites, alcohol, drugs, autoimmune diseases, and metabolic diseases [1]. The most common causes of hepatitis are viruses; namely hepatitis A, B, C, D and E viruses. Among these, hepatitis B virus (HBV) and hepatitis C virus (HCV) are the most important causes of viral hepatitis [2].

Both HBV and HCV can be transmitted through sexual, blood or vertically from mother-to-child [3]. Thus, individuals who need blood transfusion, those having multiple sexual partners and infants born from HBV or HCV infected mothers are at a high-risk for acquiring HBV or HCV infection [4]. Both viruses can cause acute and chronic infection of the liver [5, 6]. Chronic HBV and HCV infections are the leading causes of liver-related morbidity and mortality [7, 8]. Between 15% and 40% of chronically infected individuals can develop serious liver disease and transmit the viruses to others [9, 10]. Globally, around 257 million people were living with HBV infection, and 71 million people were living with HCV infection in 2015 [4, 11]. About 1 million people die each year from cases related to viral hepatitis [4]. An estimated 50% to 80% of cases of primary liver cancer result from infection with HBV [12, 13].

A safe and effective vaccine for HBV has been available since 1982, whereas no vaccine exists for HCV [14]. Treatment options for both viruses are advancing rapidly, and several new antiviral drugs have become available [15]. By the end of 2015, only 9% of HBV-infected people and 20% of HCV-infected people had been diagnosed. Of those 1.7 million people who found positive for HBV infection, only 8% were treated, while only 7% were treated among 1.1 million people who were positive for HCV infection in 2015 [4]. The global targets for 2030 are to diagnose 90% of people with HBV and HCV infections and treat 80% of eligible patients [16].

In Ethiopia, more than 60% of chronic liver disease and up to 80% of hepatocellular carcinoma (HCC) are due to chronic HBV and HCV infections [17]. According to WHO, Ethiopia is among hepatitis endemic countries in the world with intermediate to hyperendemic endemicity level [18]. However; Ethiopia is regarded as a country with no national strategy for surveillance, prevention and control of viral hepatitis. Above all Ethiopian children including children in our current study site have not had access to vaccination against HBV. Data on the epidemiology of HBV and HCV infections in Ethiopia at the community level are scarce. Particularly, HCV infection remains under-diagnosed and under-reported, despite its high infectious nature. Thus, lack of adequate epidemiological data at the community level on hepatitis in Ethiopia can affect the global targets to control HBV and HCV infections. For this reason, assessing the sero-prevalence of HBV and HCV infections at community level is very crucial to develop strategies to reduce transmission among the community members. Therefore, this study was aimed at investigating the sero-prevalence and associated factors for HBV and HCV infections among HBV unvaccinated community members in South Omo Zone, Southern Ethiopia.

## Materials and methods

### Study design, period and setting

A community-based cross-sectional study was conducted in three districts (Hamer, Debub Ari and Bena-Tsemay) of South Omo Zone, Southern Ethiopia from March to May 2018. The study area is located about 750 Kilometers from Addis Ababa, the capital city of Ethiopia. According to the 2007 Ethiopian census, the total population of South Omo Zone is 647,655 [19] and the eight largest ethnic groups are Ari (44.59%), Male (13.63%), Dasenech (8.17%), Hamer (8.01%), Bena (4.42%), Amhara (4.21%), Tsemai (3.39%), and Nyangatom (2.95%) [20]. Most of the inhabitants are nomadic pastoralists in the Hamer District and farmers in the Debub Ari and Bena-Tsemay districts [19]. The study districts were purposely selected because of their endemicity for yellow fever outbreaks [21], which has similar clinical presentations with viral hepatitis.

### Study participants, sample size estimation and sampling method

The study participants were individual’s age between 6 and 80 years old, residents of the study districts and volunteered to participate in the study. Sample size for the nomadic population (Hamer District) was calculated with the estimated HBsAg sero-prevalence of 14.5% [22] with 95% confidence, 5% margin of error, 15% non-response rate and 1.5 design effects. For the mixed farming and settled farming population (Debub Ari and Bena-Tsemay Districts) the sample size was calculated with the assumptions: sero-prevalence of HBsAg in the community is 10.5% [23] at confidence level of 95% and 5% margin of error; estimated non-response rate 10% and design effect of 2.0. Accordingly, these resulted in minimum sample size of 621. Including few extra samples collected during the data collection, the sample size on which the current study based on is 625 (306 from Hammer District and 319 from Debub Ari and Bena Tsemay Districts). A representative sample was drawn from each study kebeles (smaller administrative units) of the study districts by distributing the overall sample size proportionally to the size of population in each study kebeles. Once after the first household was selected randomly by using lottery method, the other households from each study kebeles were selected using systematic random sampling technique after getting the n^th^ value (sampling interval) by dividing the total number of households by the sample size allocated for each kebeles. From each selected households one participant was randomly recruited using lottery method.

### Data collection

Three milliliter of a blood sample was collected from each of the study participants, and serum was separated and stored at appropriate temperature until screened for HBV and HCV infections. Structured questionnaire was administered to collect socio-demographic and relevant clinical data. One step HBsAg test strip (Nantong Diagnos Technology Co., Ltd., China) was used for the screening hepatitis B surface antigen (HBsAg). Whereas one step HCV test strip (Nantong Diagnos Technology Co., Ltd., China) was used to detect antibodies against HCV following the instructions of the manufacturer. Samples found positive for HBV infection by one step HBsAg test strip were further screened using qualitative immunoassay Alere Determine^TM^ HBsAg (Alere Inc., Massachusetts, USA) which is more specific but less sensitive than the One Step HBsAg test [24–26]

### Data entry and analysis

Data were entered using Epi-Data Entry version 3.1 and analyzed using SPSS version 25.0. Descriptive statistics; mean, and standard deviation for continuous variables and frequency for categorical variables were used. Bivariable and multivariable logistic regression analysis were used to assess factors associated with sero-prevalence for HBV and HCV infections. Variables which showed association in multivariable analysis were considered as final predictors of the dependent variable. All tests were performed at 5% level of significance.

### Ethical considerations

The study was carried out after getting ethical approval from the Institutional Review Board (IRB) of Aklilu Lemma Institute of Pathobiology, Addis Ababa University. Then, data were collected after getting permission from South Omo Zone and district health offices. The objective, the possible risks & benefits of the study were explained to the participants or their guardians in local languages and informed written consent was obtained from the participants or from their guardians. For illiterate participants who not able to read and write a fingerprint were taken instead of their signature after informing and elaborating the issues written on the consent form. The participants were assured that they had full right to participate or not to participate in the study. Sero-positive individuals were advised and linked to health facilities to obtain appropriate treatment and care. All information obtained in this study was kept confidential.

## Results

### Socio-demographic characteristics of the study participants

A total of 625 participants (51.4% males, age range from 6 to 80 and mean age ± SD = 30.83 ± 13.51 years) participated in this study. Table 1 shows the socio-demographic characteristics of the study participants.

**Table 1:** Socio-demographic characteristics of the study participants.

Characteristics	Category	No. (%)
Sex	Male	321(51.4)
	Female	304(48.6)
Age group	<18	64(10.2)
	18–29	248(39.7)
	30–45	239(38.2)
	46–65	59(9.4)
	>65	15(2.4)
Religion	Orthodox	70(11.2)
	Protestant	257(41.1)
	Traditional	294(47.0)
	Other	4(0.6)
Educational status	Not attended formal education	391(62.6)
	Primary school attended	234(37.4)
Occupation	Farmer	259(41.4)
	Agro pastoralist	41(6.6)
	Nomadic pastoralist	262(41.9)
	Others	63(10.1)
District	Debub Ari	279(44.6)
	Bena-Tsemay	40(6.4)
	Hamer	306(49.0)

### Clinical sign and symptoms reported by the study participants

As shown in Table 2, the most common sign and symptoms reported were upper abdominal pain especially on the right side (18.9%) followed by joint pain and muscle aches (16.0%) and weakness and fatigue (7.5%).

**Table 2:** Sign and symptoms reported by the study participants.

Characteristics	Category	No (%)
Upper abdominal pain	Yes	118(18.9)
	No	507(81.1)
Dark urine	Yes	43(6.9)
	No	582(93.1)
Joint pain and muscle aches	Yes	100(16.0)
	No	525(84.0)
Loss of appetite	Yes	17(2.7)
	No	608(97.3)
Nausea and vomiting	Yes	26(4.2)
	No	599(95.8)
Weakness and fatigue	Yes	47(7.5)
	No	578(92.5)
Yellowing of skin and eyes (jaundice)	Yes	11(1.8)
	No	614(98.2)
Other non-specific symptoms such as fever, headache and body rash	Yes	14(2.2)
	No	611(97.8)

### Sero-prevalence for HBV and HCV infections

The overall sero-prevalence for HBV infection was 8.0% (95% CI: 5.9%, 10.2%) using one step HBsAg test strip and 7.2% (95% CI: 5.2%, 9.3%) using HBsAg Alere Determine^TM^ test. Among 50 samples found positive for HBV infection by one step HBsAg test strip, 5 samples were found negative by HBsAg Alere Determine^TM^ test. The overall sero-prevalence for HCV infection was 1.9% (95% CI: 0.9%, 3.0%). Two (0.3%) of the participants were co-infected with both HBV and HCV. Relatively higher sero-prevalence of HBsAg (10.0%) and anti-HCV (5.0%) was observed in Bena-Tsemay district. Under the bivariable analysis, HBV infection was significantly associated with weakness and fatigue (P<0.01) and HCV infection associated with age group (P = 0.02) (Table 3).

**Table 3:** Sero-prevalence for HBV and HCV infections.

Characteristics	Category	HBsAg (using Alere Determine^TM^ test)		P value	Anti-HCV		P value
		Positive (%)	Negative (%)		Positive (%)	Negative (%)	
Sex	Male	28(8.8)	290(91.2)	0.12	7(2.2)	311(97.8)	0.61
	Female	17(5.6)	287(94.4)		5(1.6)	299(98.4)	
Age Group	<18	2(3.1)	62(96.9)	0.64	1(1.6)	63(98.4)	0.02*
	18–29	17(6.9)	230(93.1)		2(0.8)	245(99.2)	
	30–45	21(8.8)	218(91.2)		4(1.7)	235(98.3)	
	46–65	4(7.0)	53(93.0)		4(7.0)	53(93.0)	
	>65	1(6.7)	14(93.3)		1(6.7)	14(93.3)	
Educational Status	Not attended formal education	26(6.7)	363(93.3)	0.49	8(2.1)	381(97.9)	0.77
	Primary school attended	19(8.2)	214(91.8)		4(1.7)	229(98.3)	
Occupation	Farmer	18(7.0)	238(93.0)	0.97	3(1.2)	253(98.8)	0.47
	Pastoralist	22(7.3)	281(92.7)		7(2.3)	296(97.7)	
	Others	5(7.9)	58(92.1)		2(3.2)	61(96.8)	
District	Debub Ari	19(6.9)	257(93.1)	0.78	2(0.7)	274(99.3)	0.09
	Bena-Tsemay	4(10.0)	36(90.0)		2(5.0)	38(95.0)	
	Hamer	22(7.2)	284(92.8)		8(2.6)	298(97.4)	
Upper abdominal pain	Yes	12(10.2)	106(89.8)	0.17	4(3.4)	114(96.6)	0.20
	No	33(6.5)	471(93.5)		8(1.6)	496(98.4)	
Dark urine	Yes	6(14.0)	37(86.0)	0.08	1(2.3)	42(97.7)	0.85
	No	39(6.7)	540(93.3)		11(1.9)	568(98.1)	
Joint pain and Muscle aches	Yes	9(9.0)	91(91.0)	0.46	2(2.0)	98(98.0)	0.96
	No	36(6.9)	486(93.1)		10(1.9)	512(98.1)	
Weakness and fatigue	Yes	9(19.1)	38(80.9)	<0.01*	0	47(100.0)	0.32
	No	36(6.3)	539(93.7)		12(2.1)	563(97.9)	
Presence of Jaundice	Yes	0	11(100.0)	0.35	1(9.1)	10(90.9)	0.08
	No	45(7.4)	566(92.6)		11(1.8)	600(98.2)	

*Significant at P value <0.05

### Independent predictors of HBV and HCV infections

A multivariable logistic regression analyses was performed to explore the independent predictors of HBV and HCV infections. Having body weakness and fatigue (AOR = 5.20; 95% CI: 1.58, 17.15) and age group 46–65 (AOR = 13.02; 95% CI: 1.11, 152.41) were significantly associated with HBV (Table 4) and HCV infections respectively at P value <0.05 (Table 5).

**Table 4:** Independent predictors of HBV infection.

Characteristics	Category	HBsAg	
		COR (95% CI)	AOR (95% CI)
**Sex**	Male	1.63(0.87, 3.04)	1.45(0.75, 2.79)
	Female	1.00	1.00
**Age Group**	<18	1.00	1.00
	18–29	2.29(0.52, 10.19)	1.51(0.31, 7.43)
	30–45	2.99(0.68, 13.09)	2.10(0.42, 10.49)
	46–65	2.34(0.41, 13.28)	1.92(0.30, 12.50)
	>65	2.21(0.19, 26.17)	1.52(0.12, 20.07)
**Educational Status**	Not attended formal education	0.81(0.44, 1.49)	0.79(0.38, 1.68)
	Primary school attended	1.00	1.00
**Occupation**	Farmer	1.00	1.00
	Pastoralist	1.04(0.54, 1.98)	1.01(0.31, 3.30)
	Others	1.14(0.41, 3.20)	1.33?(0.3, 5.08)
**District**	Debub Ari	1.00	1.00
	Bena-Tsemay	1.50(0.48, 4.67)	1.24(0.33, 4.76)
	Hamer	1.43(0.47,4.40)	1.87(0.45, 7.78)
**Upper abdominal pain**	Yes	1.62(0.8, 13.23)	1.63(0.62, 4.27)
	No	1.00	1.00
**Dark urine**	Yes	2.25(0.89, 5.64)	1.51(0.44, 5.20)
	No	1.00	1.00
**Joint pain and Muscle aches**	Yes	1.34(0.62, 2.87)	2.03(0.58, 7.05)
	No	1.00	1.00
**Weakness and fatigue**	Yes	3.55(1.59, 7.900	5.20(1.58, 17.15)*
	No	1.00	1.00

CI (confidence interval), COR (crude odds ratio), AOR (adjusted odds ratio) and *(significant at p<0.05)

**Table 5:** Independent predictors of HCV infection.

Characteristics	Category	Anti-HCV	
		COR (95% CI)	AOR (95% CI)
**Sex**	Male	1.35(0.42, 4.29)	1.15(0.33,4.02)
	Female	1.00	1.00
**Age Group**	<18	1.00	1.00
	18–29	0.51(0.05, 5.76)	0.59(0.05, 7.48)
	30–45	1.07(0.12, 9.76)	2.14(0.20, 23.28)
	46–65	4.76(0.52, 43.85)	13.02(1.11, 152.41)*
	>65	4.50(0.27, 76.38)	4.62(0.16, 137.01)
**Educational Status**	Not attended formal education	1.20(0.3, 64.04)	1.59(0.34,7.49)
	Primary school attended	1.00	1.00
**Occupation**	Farmer	1.00	1.00
	Pastoralist	1.99(0.51, 7.79)	3.81(0.24, 60.11)
	Others	2.77(0.45, 16.91)	1.36(0.05, 36.52)
**District**	Debub Ari	1.00	1.00
	Bena-Tsemay	7.21(2.00, 52.70)	11.20(1.07, 117.27)
	Hamer	1.96(0.40, 9.57)	18.20(0.65, 513.25)
Upper abdominal pain	Yes	2.18(0.64, 7.35)	1.67(0.36, 7.72)
	No	1.00	1.00
Dark urine	Yes	1.23(0.16, 9.75)	1.47(0.13,16.22)
	No	1.00	1.00
Joint pain and Muscle aches	Yes	1.05(0.23, 4.84)	2.26(0.37,13.69)
	No	1.00	1.00
Presence of Jaundice	Yes	5.46(0.64,46.38)	3.50(0.24, 52.29)
	No	1.00	1.00

CI (confidence interval), COR (crude odds ratio), AOR (adjusted odds ratio) and *(significant at p<0.05)

## Discussion

This study revealed higher-intermediate endemicity level [27, 28] of HBV infection with overall sero-prevalence of 7.2% among the general population in South Omo Zone. The sero-prevalence of HBsAg is almost similar with the previous national pooled prevalence of 7.4% of Ethiopia [23] and within the range of a prevalence of 5–10% reported among adult population in sub-Saharan African countries [29]. However the prevalence is relatively higher than 3.1% prevalence reported from a recent community-based study done in Gojjam, Northwest Ethiopia [30]; 6.1% the whole African region and 3.5% global prevalence among the general population [4]. There is a geographical variation in the sero-prevalence of HBsAg in Ethiopia with relatively higher prevalence observed in the current study area as compared to the Gojam area study. Thus the relative increase in the prevalence of HBsAg in South Omo Zone as compared to the other geographic areas suggests the current study area is one among the priority target areas for the prevention and control of hepatitis in Ethiopia.

In the case of HCV sero-prevalence, anti-HCV detection rate varied from low to higher–intermediate endemicity levels [27, 28] among the different districts with the highest sero-prevalence (5.0%) detected in Bena-Tsemay district. The overall sero-prevalence of anti-HCV in the study area was 1.9%. The overall sero-prevalence of anti-HCV recorded in our study area was less than that from the pooled national prevalence of 3.1% [23] and 3.0% reported in Sub-Saharan Africa [31]. However, it is still greater than the 1.0% prevalence reported from Gojjam, Ethiopia [30]; 0.3% in Djibouti, 0.9% in Somalia, and 1.0% in Sudan [32] among the general populations. Although the overall sero-prevalence of HCV infection in the study area is considered to be low according to the WHO classification [27, 28], relatively higher prevalence detected in Bena-Tsemay district indicates it is a marked public health problem in that district.

Since HBV and HCV share common route of transmission, co-infection between the two viruses is common, especially in high endemic areas and among people at high-risk of parenteral infection [33]. In this study co-infection between HBV and HCV infection, serologic markers was 0.3% among the general population. This is in contrast to some studies from Ethiopia where no serologic markers for HBV and HCV co-infection was reported [34–36], indicating that co-infection between HBV and HCV may not be uncommon in the areas where both HBV and HCV are endemic. Our finding is supported by many other studies conducted elsewhere which reported dual infection of HBV and HCV: 0.7% in Nigeria among prison inmates[37]; 5.9% and 2.0% in India among patients with chronic liver disease [38] and hemodialysis patients [33] respectively and 7.7% in Mongolia among patients with chronic liver disease [39]. In this study to detect the sero-prevalence of HBV infection we used the viral antigen (HBsAg) as a serologic marker while for HCV we used antibody (anti-HCV antibody). With this regard recent viral transmission may relatively contribute to high sero-prevalence of HBsAg as compared to anti-HCV which ultimately may dilute the effect in co-infection rate. Thus unlike the other studies, the observed low co-infection rate between HBV and HCV in current study area might be attributed to a more recent transmission through the sexual route in the case of HBV in addition to the expected a geographical variation.

Under the multivariate analysis, having body weakness and fatigue was independently associated with HBV serological marker (HBsAg) exposure status. Those participants having body weakness and fatigue were almost five times at higher risk of being positive for HBsAg as compared to their counterparts without such problem. Previously numerous studies revealed that fatigue (generalized body weakness) is the most commonly reported symptom in patients with viral hepatitis [40–43]. Worth noting here, however, since a good number of clinical symptoms of viral hepatitis overlap with those of arboviral infections [44], involvement of the later cannot be overruled. In fact, the study area is among the known hot spots for yellow fever outbreaks [21] and is also considered to be an endemic site for many arboviral diseases because of its proximity to neighboring country Kenya, where repeated arboviral disease outbreaks have been reported [45]. Moreover, multivariate analysis also indicated that those of participants in the age group 46 to 65 years were more than thirteen times at higher risk of being positive for HCV infection as compared to those in the age group less than 18 years. These implying, older individuals are at higher risk of getting HCV infection as compared to younger. The observed significantly higher sero-prevalence in older individuals might be attributed to frequency of exposure. Similar findings were reported from studies done in: Rwanda [46], Egypt [47] and Madagascar [48]. But here we couldn’t rule out a cohort effect related to historical campaigns of parenteral treatment or vaccination which probably increased the sero-prevalence of ant-HCV infection in older individuals (cohort of older participants) as observed in studies conducted in Egypt [49] and Cameroon [50].

### Limitations of the study

The findings of this study should be interpreted in the light of the following potential limitations. Firstly, our serologic assays for HBV and HCV detection did not provide evidence of active viremia and identification of infected individuals in the antibody-negative phase (those in the window period) for HCV detection. On the top, due to constraint of resource, we didn’t conduct confirmatory test using specific assays for samples being positive for HCV, that probably may overestimated the sero-prevalence pertaining to HCV.

## Conclusion

In general, the finding in this study revealed a higher-intermediate HBV and a low to intermediate HCV infection endemicity levels among the general population in South Omo Zone. Those individuals having body weakness and fatigue and older individuals (age group 46–65) were at higher risk of acquiring HBV and HCV infections, respectively. These observations call for responsible health policy makers to develop a practical plan of intervention with the goal of prevention and control of these infections, such as screening those belonging to the high-risk group among the general population, improvement in the expansion of HBV vaccination and provision of health education. Further research using molecular and other more sensitive and specific assays for detecting active HBV and HCV infections is also needed for the future.

## Supporting information

S1 Dataset (SAV)

## References

[1] WHOa. World Health Organization: hepatitis 2016:available at http://www.who.int/topics/hepatitis/en/ (accessed March 23, 2018).

[2] El-Serag HB. Epidemiology of viral hepatitis and hepatocellular carcinoma. Gastroenterology. 2012 5;142(6):1264–73.e1. 10.1053/j.gastro.2011.12.061 . Pubmed Central PMCID: PMC3338949. Epub 2012/04/28. eng. 22537432 PMC3338949

[3] Wasley A, Grytdal S, Gallagher K. Surveillance for acute viral hepatitis—United States, 2006. Morbidity and mortality weekly report Surveillance summaries (Washington, DC: 2002). 2008 3 21;57(2):1–24. . Epub 2008/03/21. eng. 18354374

[4] WHOa. World Health Organization: Global hepatitis report, Geneva. 2017:available at www.who.int/hepatitis/publications/globalhepatitis-report2017/en/ (accessed November 14, 8)

[5] Martin P, Lau DT, Nguyen MH, Janssen HL, Dieterich DT, Peters MG, et al A Treatment Algorithm for the Management of Chronic Hepatitis B Virus Infection in the United States: 2015 Update. Clinical gastroenterology and hepatology: the official clinical practice journal of the American Gastroenterological Association. 2015 11;13(12):2071–87.e16. 10.1016/j.cgh.2015.07.007 . Epub 2015/07/19. eng. 26188135

[6] Moyer VA. Screening for hepatitis C virus infection in adults: U.S. Preventive Services Task Force recommendation statement. Annals of internal medicine. 2013 9 3;159(5):349–57. 10.7326/0003-4819-159-5-201309030-00672 . Epub 2013/06/26. eng. 23798026

[7] El Khoury AC, Wallace C, Klimack WK, Razavi H. Economic burden of hepatitis C-associated diseases: Europe, Asia Pacific, and the Americas. Journal of medical economics. 2012;15(5):887–96. 10.3111/13696998.2012.681332 . Epub 2012/03/31. eng. 22458755

[8] Shire NJ, Sherman KE. Epidemiology of Hepatitis C Virus: A Battle on New Frontiers. Gastroenterology clinics of North America. 2015 12;44(4):699–716. 10.1016/j.gtc.2015.07.002 . Epub 2015/11/26. eng. 26600215

[9] Lok AS. Chronic hepatitis B. The New England journal of medicine. 2002 5 30;346(22):1682–3. 10.1056/NEJM200205303462202 . Epub 2002/05/31. eng. 12037146

[10] Seeff LB. Natural history of chronic hepatitis C. Hepatology (Baltimore, Md). 2002 11;36(5 Suppl 1):S35–46. 10.1053/jhep.2002.36806 . Epub 2002/10/31. eng. 12407575

[11] WHOb. World Health Organization: Hepatitis B fact sheet 2017:available at http://www.who.int/mediacentre/factsheets/fs204/en/index.html (accessed June, 2018).

[12] Perz JF, Armstrong GL, Farrington LA, Hutin YJ, Bell BP. The contributions of hepatitis B virus and hepatitis C virus infections to cirrhosis and primary liver cancer worldwide. Journal of hepatology. 2006 10;45(4):529–38. 10.1016/j.jhep.2006.05.013 . Epub 2006/08/02. eng. 16879891

[13] WHOa. World Health Organization: Hepatitis C 2011:available at http://ecdc.europa.eu/en/publications/Publications/TER_100914_Hep_B_C%20_EU_neighbourhood.pdf (accessed September 27, 2018)

[14] WHO. World Health Organization: Hepatitis C Virus Prevention 2018:available at: http://www.who.int/news-room/fact-sheets/detail/hepatitis-c (accessed November 18, 2018)

[15] Kohli A, Shaffer A, Sherman A, Kottilil S. Treatment of hepatitis C: a systematic review. Jama. 2014 8 13;312(6):631–40. 10.1001/jama.2014.7085 . Epub 2014/08/15. eng. 25117132

[16] WHO. World Health Organization: Combating Hepatitis B and C to Reach Elimination by 2030, Geneva, Switzerland. 2016: available at https://www.who.int/hepatitis/publications/hep-elimination-by-2030-brief/en/ (accessed September 29, 2018) 2016.

[17] Bane A, Patil A, M. K. Healthcare cost and access to care for viral hepatitis in Ethiopia. IJIAS. 2014;9(4):1718–23.

[18] WHO. Global policy report on the prevention and control of viral hepatitis. 2013:p.536 available at http://www.who.int/hiv/pub/hepatitis/global_report/en/ (accessed September 16, 2018)

[19] ONM. Organization for Natural Medicine (ONM): Lower Omo Project Area. 2017: p. avalable at http://www.onmloweromo.org/workarea.html (accessed November 22, 2018). 2017.

[20] CSAE. Central Statistical Agency Of Ethiopia: 2007 Population and Housing Census of Ethiopia: Southern Nations, Nationalities and Peoples' Region, Tables 2.1, 2.4, 2.5, 3.1, 3.2 and 3.4. 2008.

[21] Lilay A, Asamene N, Bekele A, Mengesha M, Wendabeku M, Tareke I, et al Reemergence of yellow fever in Ethiopia after 50 years, 2013: epidemiological and entomological investigations. BMC infectious diseases. 2017 5 15;17(1):343 10.1186/s12879-017-2435-4 . Pubmed Central PMCID: PMC5432991. Epub 2017/05/17. eng. 28506254 PMC5432991

[22] Taye S, Abdulkerim A, Hussen M. Prevalence of hepatitis B and C virus infections among patients with chronic hepatitis at Bereka Medical Center, Southeast Ethiopia: a retrospective study. BMC research notes. 2014 4 29;7:272 10.1186/1756-0500-7-272 . Pubmed Central PMCID: PMC4032359. Epub 2014/04/30. eng. 24774645 PMC4032359

[23] Belyhun Y, Maier M, Mulu A, Diro E, Liebert UG. Hepatitis viruses in Ethiopia: a systematic review and meta-analysis. BMC infectious diseases. 2016 12 19;16(1):761 10.1186/s12879-016-2090-1 . Pubmed Central PMCID: PMC5168848. Epub 2016/12/21. eng. 27993129 PMC5168848

[24] Chisenga CC, Musukuma K, Chilengi R, Zurcher S, Munamunungu V, Siyunda A, et al Field performance of the Determine HBsAg point-of-care test for diagnosis of hepatitis B virus co-infection among HIV patients in Zambia. Journal of clinical virology: the official publication of the Pan American Society for Clinical Virology. 2018 1;98:5–7. 10.1016/j.jcv.2017.11.005 . Pubmed Central PMCID: PMC5794713. Epub 2017/11/28. eng. 29175231 PMC5794713

[25] Shivkumar S, Peeling R, Jafari Y, Joseph L, Pai NP. Rapid point-of-care first-line screening tests for hepatitis B infection: a meta-analysis of diagnostic accuracy (1980–2010). The American journal of gastroenterology. 2012 9;107(9):1306–13. 10.1038/ajg.2012.141 . Epub 2012/05/30. eng. 22641308

[26] WHOb. World Health Organization. Hepatitis B Surface Antigen Assays: Operational Characteristics (Phase 1) Report 1. Geneva: World Health Organization; 2001. 2011.

[27] Ott JJ, Stevens GA, Groeger J, Wiersma ST. Global epidemiology of hepatitis B virus infection: new estimates of age-specific HBsAg seroprevalence and endemicity. Vaccine. 2012 3 9;30(12):2212–9. 10.1016/j.vaccine.2011.12.116 . Epub 2012/01/26. eng. 22273662

[28] Schweitzer A, Horn J, Mikolajczyk RT, Krause G, Ott JJ. Estimations of worldwide prevalence of chronic hepatitis B virus infection: a systematic review of data published between 1965 and 2013. Lancet (London, England). 2015 10 17;386(10003):1546–55. 10.1016/S0140-6736(15)61412-X . Epub 2015/08/02. eng. 26231459

[29] WHOb. World Health Organization: Hepatitis B fact sheet 2016:available at http://www.who.int/mediacentre/factsheets/fs204/en/# (accessed November 16, 2018).

[30] Abera B, Adem Y, Yimer M, Mulu W, Zenebe Y, Mekonnen Z. Community seroprevalence of hepatitis B, C and human immunodeficiency virus in adult population in gojjam zones, northwest Ethiopia. Virology journal. 2017 2 6;14(1):21 10.1186/s12985-017-0696-6 . Pubmed Central PMCID: PMC5294870. Epub 2017/02/09. eng. 28166829 PMC5294870

[31] Mora N, Adams WH, Kliethermes S, Dugas L, Balasubramanian N, Sandhu J, et al A Synthesis of Hepatitis C prevalence estimates in Sub-Saharan Africa: 2000–2013. BMC infectious diseases. 2016 6 13;16:283 10.1186/s12879-016-1584-1 . Pubmed Central PMCID: PMC4906983. Epub 2016/06/15. eng. 27296465 PMC4906983

[32] Chaabna K, Kouyoumjian SP, Abu-Raddad LJ. Hepatitis C Virus Epidemiology in Djibouti, Somalia, Sudan, and Yemen: Systematic Review and Meta-Analysis. PloS one. 2016;11(2):e0149966 10.1371/journal.pone.0149966 . Pubmed Central PMCID: PMC4764686. Epub 2016/02/24. eng. 26900839 PMC4764686

[33] Malhotra R, Soin D, Grover P, Galhotra S, Khutan H, Kaur N. Hepatitis B virus and hepatitis C virus co-infection in hemodialysis patients: A retrospective study from a tertiary care hospital of North India. Journal of natural science, biology, and medicine. 2016 Jan-Jun;7(1):72–4. 10.4103/0976-9668.175076 . Pubmed Central PMCID: PMC4780172. Epub 2016/03/24. eng. 27003974 PMC4780172

[34] Birku T, Gelaw B, Moges F, Assefa A. Prevalence of hepatitis B and C viruses infection among military personnel at Bahir Dar Armed Forces General Hospital, Ethiopia. BMC research notes. 2015 12 1;8:737 10.1186/s13104-015-1719-2 . Pubmed Central PMCID: PMC4666071. Epub 2015/12/03. eng. 26625733 PMC4666071

[35] Gelaw B, Y M. Prevalence of HBV, HCV and malaria parasiets among blood donors in Amhara and tigray regional states. Ethiop J Health Dev 2007;2(22):3–7.

[36] Molla S, Munshea A, Nibret E. Seroprevalence of hepatitis B surface antigen and anti HCV antibody and its associated risk factors among pregnant women attending maternity ward of Felege Hiwot Referral Hospital, northwest Ethiopia: a cross-sectional study. Virology journal. 2015 12 2;12:204 10.1186/s12985-015-0437-7 . Pubmed Central PMCID: PMC4667425. Epub 2015/12/03. eng. 26626263 PMC4667425

[37] Adoga MP, Banwat EB, Forbi JC, Nimzing L, Pam CR, Gyar SD, et al Human immunonodeficiency virus, hepatitis B virus and hepatitis C virus: sero-prevalence, co-infection and risk factors among prison inmates in Nasarawa State, Nigeria. Journal of infection in developing countries. 2009 8 30;3(7):539–47. 10.3855/jidc.472 . Epub 2009/09/19. eng. 19762972

[38] Saravanan S, Velu V, Nandakumar S, Madhavan V, Shanmugasundaram U, Murugavel KG, et al Hepatitis B virus and hepatitis C virus dual infection among patients with chronic liver disease. Journal of microbiology, immunology, and infection = Wei mian yu gan ran za zhi. 2009 4;42(2):122–8. . Epub 2009/07/15. eng. 19597643

[39] Tsatsralt-Od B, Takahashi M, Nishizawa T, Endo K, Inoue J, Okamoto H. High prevalence of dual or triple infection of hepatitis B, C, and delta viruses among patients with chronic liver disease in Mongolia. Journal of medical virology. 2005 12;77(4):491–9. 10.1002/jmv.20482 . Epub 2005/10/29. eng. 16254981

[40] Ashrafi M, Modabbernia A, Dalir M, Taslimi S, Karami M, Ostovaneh MR, et al Predictors of mental and physical health in non-cirrhotic patients with viral hepatitis: a case control study. Journal of psychosomatic research. 2012 9;73(3):218–24. 10.1016/j.jpsychores.2012.06.006 . Epub 2012/08/02. eng. 22850263

[41] Evon DM, Wahed AS, Johnson G, Khalili M, Lisker-Melman M, Fontana RJ, et al Fatigue in Patients with Chronic Hepatitis B Living in North America: Results from the Hepatitis B Research Network (HBRN). Digestive diseases and sciences. 2016 4;61(4):1186–96. 10.1007/s10620-015-4006-0 . Pubmed Central PMCID: PMC4791302. Epub 2016/02/03. eng. 26831489 PMC4791302

[42] Hann HW, Han SH, Block TM, Harris M, Maa JF, Fisher RT, et al Symptomatology and health attitudes of chronic hepatitis B patients in the USA. Journal of viral hepatitis. 2008 1;15(1):42–51. 10.1111/j.1365-2893.2007.00895.x . Pubmed Central PMCID: PMC2229833. Epub 2007/12/20. eng. 18088244 PMC2229833

[43] Karaivazoglou K, Iconomou G, Triantos C, Hyphantis T, Thomopoulos K, Lagadinou M, et al Fatigue and depressive symptoms associated with chronic viral hepatitis patients. health-related quality of life (HRQOL). Annals of hepatology. 2010 Oct-Dec;9(4):419–27. . Epub 2010/11/09. eng. 21057161

[44] WHO/PAHO. World Health Organization/Pan American Health Organization: Control of Yellow Fever, Field Guide. 2005;Washington, Scientific and Technical Publication No. 603, ISBN 92 75 11603 2

[45] Sutherland LJ, Cash AA, Huang YJ, Sang RC, Malhotra I, Moormann AM, et al Serologic evidence of arboviral infections among humans in Kenya. The American journal of tropical medicine and hygiene. 2011 7;85(1):158–61. 10.4269/ajtmh.2011.10-0203 . Pubmed Central PMCID: PMC3122361. Epub 2011/07/08. eng. 21734142 PMC3122361

[46] Umumararungu E, Ntaganda F, Kagira J, Maina N. Prevalence of Hepatitis C Virus Infection and Its Risk Factors among Patients Attending Rwanda Military Hospital, Rwanda. BioMed research international. 2017;2017:5841272 10.1155/2017/5841272 . Pubmed Central PMCID: PMC5299157. Epub 2017/03/02. eng. 28246598 PMC5299157

[47] Abdel-Aziz F, Habib M, Mohamed MK, Abdel-Hamid M, Gamil F, Madkour S, et al Hepatitis C virus (HCV) infection in a community in the Nile Delta: population description and HCV prevalence. Hepatology (Baltimore, Md). 2000 7;32(1):111–5. 10.1053/jhep.2000.8438 . Epub 2000/06/28. eng. 10869297

[48] Ramarokoto CE, Rakotomanana F, Ratsitorahina M, Raharimanga V, Razafindratsimandresy R, Randremanana R, et al Seroprevalence of hepatitis C and associated risk factors in urban areas of Antananarivo, Madagascar. BMC infectious diseases. 2008 2 29;8:25 10.1186/1471-2334-8-25 . Pubmed Central PMCID: PMC2292193. Epub 2008/03/04. eng. 18312652 PMC2292193

[49] Rao MR, Naficy AB, Darwish MA, Darwish NM, Schisterman E, Clemens JD, et al Further evidence for association of hepatitis C infection with parenteral schistosomiasis treatment in Egypt. BMC infectious diseases. 2002 Dec 4;2:29 10.1186/1471-2334-2-29 . Pubmed Central PMCID: PMC139974. Epub 2002/12/05. eng. 12464161 PMC139974

[50] Nerrienet E, Pouillot R, Lachenal G, Njouom R, Mfoupouendoun J, Bilong C, et al Hepatitis C virus infection in cameroon: A cohort-effect. Journal of medical virology. 2005 6;76(2):208–14. 10.1002/jmv.20343 . Epub 2005/04/19. eng. 15834878

